# Advanced Exergy Analysis of Adiabatic Underwater Compressed Air Energy Storage System

**DOI:** 10.3390/e25010077

**Published:** 2022-12-30

**Authors:** Lukasz Szablowski, Tatiana Morosuk

**Affiliations:** 1 Institute of Heat Engineering, Warsaw University of Technology, 00-665 Warsaw, Poland; 2 Institute for Energy Engineering, Technische Universität Berlin, 10587 Berlin, Germany

**Keywords:** energy storage, underwater compressed air energy storage, exergy analysis, advanced exergy analysis

## Abstract

Rapid development in the renewable energy sector require energy storage facilities. Currently, pumped storage power plants provide the most large-scale storage in the world. Another option for large-scale system storage is compressed air energy storage (CAES). This paper discusses a particular case of CAES—an adiabatic underwater energy storage system based on compressed air—and its evaluation using advanced exergy analysis. The energy storage system is charged during the valleys of load and discharged at peaks. The model was built using Aspen HYSYS software. Advanced exergy analysis revealed interactions between system components and the potential for improving both system components individually and the system as a whole. The most significant reduction in exergy destruction can be achieved with heat exchangers. The round-trip efficiency of this system is 64.1% and 87.9% for real and unavoidable operation conditions, respectively.

## 1. Introduction

Currently, pumped storage power plants are the leading stored energy solution in the world. Compressed air energy storage (CAES) comes a distant second.

The CAES solution is characterized by low-density stored energy, requiring large-volume compressed air tanks. Diabatic CAES is a well-known, mature technology. There are just two large power plants of this type in the world, as follows:

- Huntorf power plant (Germany) was constructed in 1978 with an installed capacity of 290 MW, later upgraded up to 321 MW;
- McIntosh power plant (USA) was constructed in 1991 with an installed capacity of 110 MW.

A review of CAES technology can be found in [1,2,3,4,5]. A hybrid system consisting of CAES cooperating with renewable energy sources and potential locations in Poland is dealt with in detail in [6]. Dynamic mathematical models of CAES systems are presented in [6,7,8,9,10].

Whereas a constant storage volume characterizes the above-described systems, an alternative to them may be found in the form of constant pressure systems. One such example is the underwater compressed air energy storage system (UWCAES), which uses special underwater balloons for compressed air. The pressure of the stored air depends here on the depth at which the underwater bags are placed.

The impact of design parameters on the efficiency of the UWCAES system is described in [11]. This system proved to be very sensitive to the compressor and turbine efficiency and the depth at which the air reservoirs were installed.

Numerical calculations using the multi-objective optimization method via a genetic algorithm of the UWCAES system with a capacity of 4 MWh are presented in [12].

Airbags for storage of compressed air underwater were experimentally tested in [13]. Initially, balloons with a diameter of 1.8 m were placed underwater and subjected to 400 loading and unloading cycles. Then, balloons with a diameter of 5 m were placed at a depth of 25 m. It was confirmed that it is possible to store compressed air underwater (even sea water) using appropriate materials.

Simulation of water flow around the UWCAES compressed air balloons using numerical methods of fluid dynamics is presented in [14].

Furthermore, [15] describes a hybrid UWCAES system that can also generate electricity from tidal flows (VIVACE, or vortex-induced vibration aquatic clean energy). In this system, compressed air tanks play the role of movable floats.

A simulation of the impact of loads caused by water flow on underwater air reservoirs is presented in [16].

An isothermal UWCAES system was proposed in [17]. It was achieved by using a hydro-pneumatic system. Part of the system, responsible for energy conversion, was set on a floating platform.

In [18], a dynamic model of the adiabatic UWCAES system was built. Despite constant pressure in the air-filled tank, the model is dynamic due to the simulation of the model’s cooperation with the electrical grid. Exergy balances were carried out for the model.

Liquid air can be substituted in to provide an alternative to systems that use compressed air for energy storage [19].

Conventional and advanced exergy analysis of adiabatic underwater compressed air energy storage systems were reported in [20,21]. In these works, two approaches to the issue of pressure in airbags were proposed, namely, in [20], variable pressure in bags (which would mean moving bags up and down), and, in [21], throttling just before the air reservoir to maintain a constant pressure. These approaches raise some controversy. Indeed, [20] describes theoretical considerations and, therefore, changes in the depth of the location of flexible reservoirs could somehow be explained. In [21], the existing UWCAES system was described. The compression ratio of each compressor did not change despite lower requirements for this parameter (due to lower losses in heat exchangers in unavoidable and ideal conditions), which was hidden by attributing greater pressure losses to the pipeline just before the air reservoir. Moreover, the more ideal the system was, the more losses were assigned to the pipeline before air bags (which seems contradictory).

In this paper, the authors conducted the advanced exergy analysis of an adiabatic underwater compressed air energy storage system using the procedure with constant pressure in the air reservoir (located at the same depth), but without artificial throttling (for real, unavoidable, and ideal conditions). This means that larger pressure drops on heat exchangers will force larger increases for individual parts of the compressor and smaller drops for individual parts of the turbine.

## 2. Materials and Methods

The round-trip efficiency of compressed air energy storage systems is described by the following definition:

(1) $$\eta_{CAES}=\frac{EN_{g}}{EN_{c}+EN_{f}}$$

where *EN_g_* is energy transferred by work to the generator (J), *EN_c_* is energy consumed by a compressor in the form of work (J), and *EN_f_* is the chemical energy of a fuel (J) (0 in adiabatic systems). The chemical energy of the fuel can be calculated by multiplying the amount of fuel used by its lower heating value. In the case discussed in this article, fuel is not used; therefore, the above formula takes into account only *EN_g_* and *EN_c_*.

### 2.1. Conventional Exergy Analysis

The exergy balance for a system is as follows:

(2) $${\dot{E}}_{F,tot}={\dot{E}}_{P,tot}+{\dot{E}}_{D,tot}+{\dot{E}}_{L,tot}={\dot{E}}_{P,tot}+\underset{n}{\sum }{\dot{E}}_{D,k}+{\dot{E}}_{L,tot}$$

where ${\dot{E}}_{F,tot}$ is the exergy of “fuel” for the overall system (W), ${\dot{E}}_{P,tot}$ is the exergy of the product for the overall system (W), ${\dot{E}}_{D,tot}$ is the total exergy destruction (W), ${\dot{E}}_{L,tot}$ are total exergy losses (W), and ${\dot{E}}_{D,k}$ is the exergy destruction in the *k*-th system component (W).

The exergy balance for the *k*-th component is as follows:

(3) $${\dot{E}}_{F,k}={\dot{E}}_{P,k}+{\dot{E}}_{D,k}$$

where ${\dot{E}}_{F,k}$ is the exergy of “fuel” for the *k*-th component (W), and ${\dot{E}}_{P,k}$ is the exergy of “product” for the *k*-th component (W).

The exergy efficiency of the system is calculated as follows:

(4) $$\varepsilon_{tot}=\frac{{\dot{E}}_{P,tot}}{{\dot{E}}_{F,tot}}$$

while exergy efficiency of the *k*-th component is calculated as follows:

(5) $$\varepsilon_{k}=\frac{{\dot{E}}_{P,k}}{{\dot{E}}_{F,k}}$$

The share of exergy destruction in the *k*-th component is determined as follows:

(6) $$y_{D,k}=\frac{{\dot{E}}_{D,k}}{{\dot{E}}_{F,tot}}$$

The share of exergy loss in the entire system is calculated as follows:

(7) $$y_{L}=\frac{{\dot{E}}_{L,tot}}{{\dot{E}}_{F,tot}}$$

More about conventional exergy analysis can be found in [22].

### 2.2. Advanced Exergy Analysis

The exergy balance may be discussed in a more detailed way. According to the definition of the advanced exergy analysis [22,23], the exergy destruction in each component can be divided into (i) endogenous/exogenous and (ii) avoidable/unavoidable.

The endogenous exergy destruction of a component should be calculated at the assumption that all system components are ideal and that the component under consideration has its real efficiency. This means that to calculate endogenous exergy destruction for all components of the system, it is necessary to build as many models of the considered system as the number of the system components. Exogenous exergy destruction is calculated by subtracting the endogenous exergy destruction of a component from its total exergy destruction. Thus, the exergy destruction in the *k*-th component can be described as a sum, as follows:

(8) $${\dot{E}}_{D,k}={\dot{E}}_{D,k}^{EN}+{\dot{E}}_{D,k}^{EX}$$

where ${\dot{E}}_{D,k}^{EN}$ is endogenous exergy destruction in the *k*-th component (W), and ${\dot{E}}_{D,k}^{EX}$ is exogenous exergy destruction in the *k*-th component (W).

The value of the exergy destruction can also be divided into avoidable and unavoidable parts, as follows:

(9) $${\dot{E}}_{D,k}={\dot{E}}_{D,k}^{UN}+{\dot{E}}_{D,k}^{AV}$$

where ${\dot{E}}_{D,k}^{UN}$ is unavoidable exergy destruction in the *k*-th component (W), and ${\dot{E}}_{D,k}^{AV}$ is avoidable exergy destruction in the *k*-th component (W).

Unavoidable exergy destruction is a part that cannot be reduced due to technological and economic limits. Thus, this is the part of the exergy destruction that will be generated in the component when it is the best in terms of currently available technology. More about advanced exergy analysis, evaluation criteria, and methodology can be found in [24,25,26].

## 3. Description of the Model

**Figure 1:**
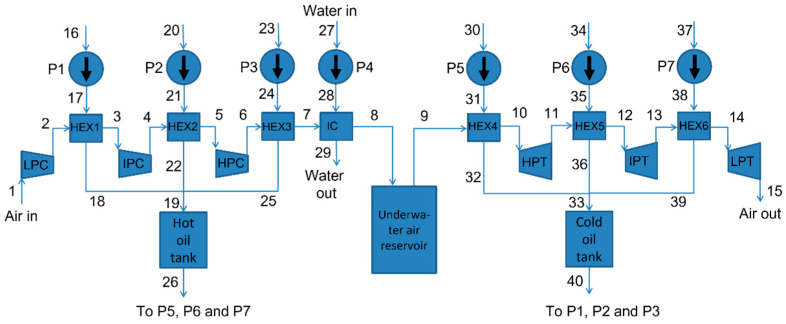
Flow diagram of adiabatic UWCAES. Abbreviations are as follows: LPC, IPC, and HPC—low-, intermediate-, and high-pressure compressor, respectively; LPT, IPT, and HPT—low-, intermediate-, and high-pressure turbine, respectively; HEX—heat exchanger; IC—intercooler; P—pump.

The UWCAES systems described in this article were modeled using Aspen HYSYS software. A schematic diagram of the modeled adiabatic UWCAES system is shown in Figure 1.

The intake air composition/concentration is given in Table 1; it is assumed to have a temperature of 15 °C and a pressure of 1 bar. During low demand for electricity, it is stored in the system (in a different form). Power is released at peaks in demand. It was assumed that the charging/discharge time ratio is 2 (charging time—eight hours; discharge time—four hours).

For heat storage purposes, synthetic oil (Therminol 55 [25]) was used, which can work in the range of −28 °C ÷ 315 °C [26].

Increasing the number of heat exchangers between compressors and turbines (and dividing turbines and compressors into more parts) improves the efficiency of heat transfer from the compressor part to the turbine part but, at the same time, it is a source of additional pressure losses (on heat exchangers). For the assumed pressure losses on the heat exchangers, there is a certain optimum for the number of turbines, compressors, and heat exchangers.

The UWCAES system modeled in this article has three parts in the compressor (low, intermediate, and high pressure), and three parts in the turbine (high, intermediate, and low pressure). Behind each part of the compressor and before each part of the turbine, there is a heat exchanger powered by thermal oil to cool (behind the compressors) or to heat (before the turbines) the air. The receiving thermal oil is designed to absorb heat from the compressor part (during loading the system) and deliver it to the turbine part (unloading the system). During the charging process of the UWCAES system, the cooled thermal oil (from the cold oil tank) goes to the HEX1-3 heat exchangers and cools the air after each part of the compressor. During this process, the oil is significantly heated and stored in a hot oil tank. The pre-cooled compressed air is additionally cooled in a water-fed intercooler (IC) and then goes to the compressed air tanks (in Figure 1 this is schematically drawn as one tank).

The UWCAES systems have compressed air tanks of variable volume. As a result, the pressure in the compressed air tanks is constant and depends on the depth of installation of such a tank (flexible tank) underwater. Therefore, the pressure behind the compressor as well as just before the turbines, is constant and, thus, the mass flows (resulting from the pressure difference) are also constant.

During the unloading of the system, thermal oil from the hot oil tank feeds the HEX4-6 heat exchangers to heat the air in the turbine part of the system, thereby increasing the power received from the turbines (and energy storage efficiency). After leaving the HEX4-6 exchangers, the cooled oil goes to the cold oil tank. At this point, the entire charge–discharge cycle ends and can be repeated again.

The number of pumps is the same as the number of heat exchangers so that the oil flows can be controlled to obtain specific air temperatures behind these heat exchangers.

Storage pressure is relatively low (15 bar), which corresponds to the depth under the surface of the water—150 m. This depth can be obtained in a water reservoir, which may be achievable, for example, after the planned flooding of the strip mine excavations in the vicinity of the Belchatow power plant, Poland.

The air mass flow during charging was 100 kg/s and during discharging it was 200 kg/s. The water-feeding aftercooler (IC) has a temperature of 15 °C, while the air leaving it has a slightly higher temperature than the inlet water (depending on the effectiveness of this heat exchanger, see Table 2). Air stored underwater has a temperature of 4 °C (after a few hours in the airbag at a significant depth it reaches the temperature of the surrounding water). Other system parameters for real, unavoidable, and ideal conditions are shown in Table 2.

## 4. Results

The round-trip energy efficiency of the adiabatic UWCAES system for real and unavoidable conditions is shown in Table 3.

The definition of efficiencies is as follows:

(10) $$\eta_{UWCAES\_Gross}=\frac{EN_{g}}{EN_{c}+EN_{P1-4}}$$

where *EN*_*P*1–4_ is the energy consumed by pumps 1–4 (J), *EN_g_* is the energy transferred by work to the generator (J), and *EN_c_* is the energy consumed by a compressor in the form of work (J). Equation (11) is as follows:

(11) $$\eta_{UWCAES\_Net}=\frac{EN_{g}-EN_{P5-7}}{EN_{c}+EN_{P1-4}}$$

where *EN*_*P*5–7_ is the energy consumed by pumps 5–7 (J), *EN_g_* is the energy transferred by work to the generator (J), and *EN_c_* is the energy consumed by a compressor in the form of work (J).

The round-trip energy efficiency of the system is greatly boosted through improvements in each component, by almost 24 percentage points. The difference between gross and net efficiency is that gross efficiency does not consider the system’s needs (e.g., energy consumed by the pumps of the unloading part of the system).

The efficiency of the UWCAES system presented in Table 3 should be compared with the results presented in the latest related articles. In [27], the efficiency of the energy storage system was reported as 70.74%, and in [28] it was 55.85%. The first result is between real and unavoidable conditions calculated here, while the efficiency presented in the second article is slightly lower than the efficiency for real conditions.

### Advanced Exergy Analysis

The advanced exergy analysis was conducted at the assumption of maintaining a constant pressure in the air reservoir (located at the same depth) of 15 bar. This means that larger pressure drops on heat exchangers will force larger increases for individual parts of the compressor and smaller drops for individual parts of the turbine.

Parameters of the UWCAES system while loading in selected places for real, unavoidable, and ideal conditions are shown in Table 4. The numbering of these points has been marked in Figure 1. Parameters of the UWCAES system while unloading in selected places for real, unavoidable, and ideal conditions are shown in Table 5.

The maximum storage volume (for real conditions) of compressed air bags is 160,192 m^3^ and after reaching the temperature of the surrounding water (4 °C), the volume of airbags drops to 150,224 m^3^.

**Figure 2:**
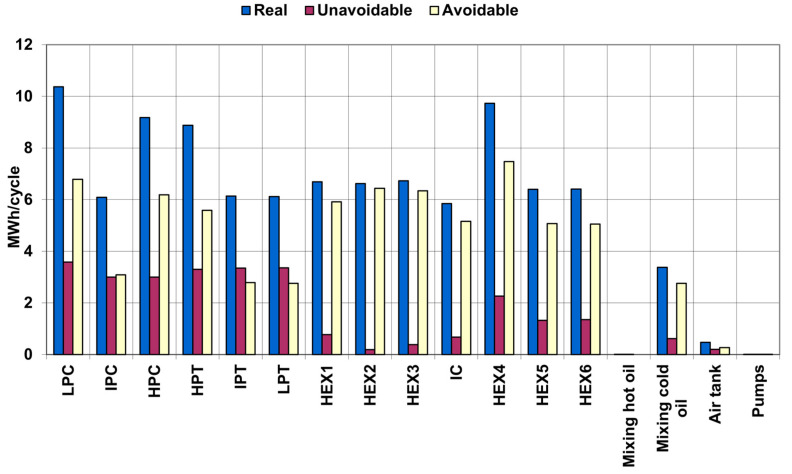
Comparison of real, unavoidable, and avoidable exergy destruction of adiabatic UWCAES.

A comparison of real, unavoidable, and avoidable exergy destruction of the adiabatic UWCAES system is shown in Figure 2. The results demonstrate the very large potential for reducing exergy destruction by improving individual system components, in particular the compressors, turbines, and heat exchangers. The greatest reduction in exergy destruction can be achieved within heat exchangers.

It is also worth analyzing the possibility of eliminating the destruction of exergy resulting from the mixing of oil of different temperatures downstream of the turbine part. This exergy destruction occurs because the air just before the HEX4 heat exchanger comes directly from the underwater flexible bag and is much cooler than the air behind the turbines. Therefore, the HEX4 heat exchanger operates under different conditions than the HEX5 and HEX6 heat exchangers, which makes the oil from this exchanger cooler than from the HEX5 and HEX6 heat exchangers.

The exergy destruction resulting from the mixing of cold oil is quite large, but the attempt to eliminate it will negatively affect the exergy destruction on the HEX4 heat exchanger by increasing the temperature differences between the factors flowing through this heat exchanger.

**Figure 3:**
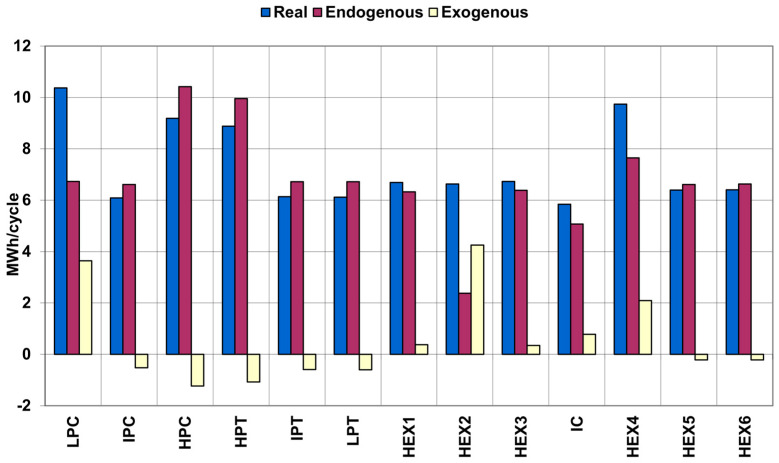
Comparison of real, endogenous, and exogenous exergy destruction of adiabatic UWCAES (except pumps).

**Figure 4:**
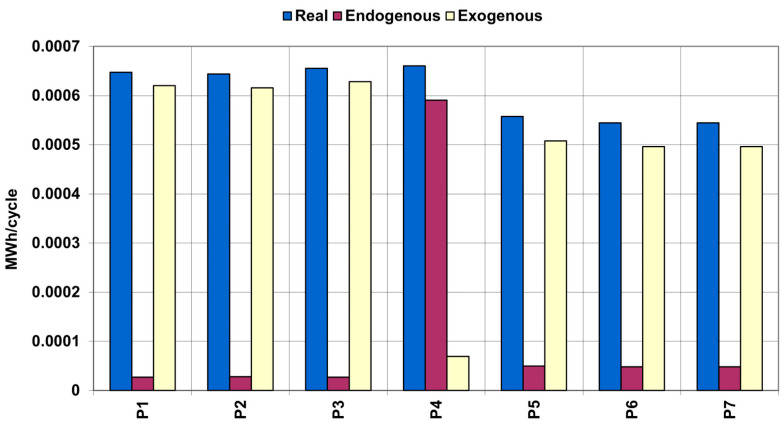
Comparison of real, endogenous, and exogenous exergy destruction of the pumps of adiabatic UWCAES.

Figure 3 shows a comparison of real, endogenous, and exogenous exergy destruction of the system (except pumps). Real, endogenous, and exogenous exergy destruction for the pumps are shown in Figure 4.

Figure 3 shows that there are system elements on which the other elements have a positive effect (reducing their exergy destruction). These elements include IPC, HPC, HPT, IPT, LPT, HEX5, and HEX6.

It is also worth noting that the remaining elements of the UWCAES system have a very negative effect on the low-pressure part of the compressor and the HEX2 heat exchanger. A noticeable negative impact of the other elements of the system is also found in the case of the HEX4 heat exchanger.

Figure 4 shows that other system components have a very negative effect on oil pumps (most likely, the negative effect is caused by imperfections of heat exchangers, such as pressure drops).

## 5. Conclusions

This article reports the application of the advanced exergy analysis to the adiabatic UWCAES system. The results show great potential for reducing exergy destruction by improving the main system components, namely compressors, turbines, and heat exchangers. The greatest reduction in exergy destruction can be achieved with heat exchangers. There are system elements on which the other elements have a positive effect (reducing their exergy destruction). These include IPC, HPC, HPT, IPT, LPT, HEX5, and HEX6.

As mentioned in the previous section, there are cases where an attempt to reduce exergy destruction on one system component can result in a significant increase in exergy destruction on another system component. An example is the destruction of exergy from mixing oil leaving the HEX4-6 heat exchangers. Eliminating (or reducing) this exergy destruction would increase the exergy destruction on the HEX4 heat exchanger, which is already large.

These analyses also show that the pumps’ exergy destruction is orders of magnitude smaller than for other system components. The round-trip energy efficiency of this system is 64.13% and 87.94% for the real and unavoidable conditions, respectively.

## Figures and Tables

**Table 1:** Composition and mass concentration of intake air.

Component	wt, %
Oxygen	23.052
Nitrogen	74.990
Carbon dioxide	0.046
Argon	1.276
Water	0.636

**Table 2:** Real, unavoidable, and ideal conditions for an adiabatic UWCAES system (based on [20,21]).

Component	Parameter	Real	Unavoidable	Ideal
LPC	Efficiency	90%	95%	100%
IPC	Efficiency	90%	95%	100%
HPC	Efficiency	85%	95%	100%
HPT	Efficiency	85%	95%	100%
IPT	Efficiency	90%	95%	100%
LPT	Efficiency	90%	95%	100%
HEX1	Effectiveness	0.9	0.98	1
	Pressure loss ratio	7.47%	1%	0%
HEX2	Effectiveness	0.9	0.98	1
	Pressure loss ratio	7.47%	1%	0%
HEX3	Effectiveness	0.9	0.98	1
	Pressure loss ratio	7.47%	1%	0%
IC	Effectiveness	0.9	0.98	1
	Pressure loss ratio	7.47%	1%	0%
HEX4	Effectiveness	0.9	0.98	1
	Pressure loss ratio	7.47%	1%	0%
HEX5	Effectiveness	0.9	0.98	1
	Pressure loss ratio	7.47%	1%	0%
HEX7	Effectiveness	0.9	0.98	1
	Pressure loss ratio	7.47%	1%	0%
Pumps	Efficiency	85%	95%	100%

**Table 3:** The round-trip energy efficiency of adiabatic UWCAES system for real and unavoidable conditions.

Efficiency	Real Conditions	Unavoidable Conditions
Gross, %	64.14	87.94
Net, %	64.13	87.94

**Table 4:** Parameters of the UWCAES system while loading in selected places for real, unavoidable, and ideal conditions.

State		Real			Unavoidable			Ideal *	
	$\dot{m}$ kg/s	*p*, bar	*T*, °C	$\dot{m}$ kg/s	*p*, bar	*T*, °C	$\dot{m}$ kg/s	*p*, bar	*T*, °C
1	100.000	1.00	15.0	100.000	1.00	15.0	100.000	1.00	15.0
2	100.000	4.09	174.8	100.000	2.79	118.2	100.000	2.49	100.2
3	100.000	3.78	73.3	100.000	2.76	29.8	100.000	2.49	15.7
4	100.000	8.64	174.9	100.000	6.52	118.2	100.000	6.12	99.8
5	100.000	8.00	73.3	100.000	6.45	29.2	100.000	6.1	15.7
6	100.000	17.51	175.6	100.000	15.29	118.2	100.000	14.99	99.9
7	100.000	16.20	73.3	100.000	15.14	29.6	100.000	14.99	15.7
8	100.000	14.99	21.1	100.000	14.99	15.3	100.000	14.99	15.0
9–15	-	-	-	-	-	-	-	-	-
16	44.621	1.00	62.0	47.923	1.00	28.0	51.801	1.00	15.7
17	44.621	1.08	62.0	47.923	1.01	28.0	51.801	1.00	15.7
18	44.621	1.00	164.4	47.923	1.00	116.5	51.801	1.00	100.2
19	134.14	1.00	165.0	149.96	1.00	116.9	155.880	1.00	100.0
20	44.373	1.00	62.0	50.696	1.00	28.0	52.941	1.00	15.7
21	44.373	1.08	62.0	50.696	1.01	28.0	52.941	1.00	15.7
22	44.373	1.00	165.4	50.696	1.00	117.5	52.941	1.00	99.8
23	45.148	1.00	62.0	51.34	1.00	28.0	51.134	1.00	15.7
24	45.148	1.08	62.0	51.34	1.01	28.0	51.134	1.00	15.7
25	45.148	1.00	165.15	51.34	1.00	116.6	51.134	1.00	99.9
26	-	-	-	-	-	-	-	-	-
27	29.247	1.00	15.0	28.528	1.00	15.0	27.006	1.00	15.0
28	29.247	1.08	15.0	28.528	1.01	15.0	27.006	1.00	15.0
29	29.247	1.00	67.6	28.528	1.00	29.3	27.006	1.00	15.7
30–39	-	-	-	-	-	-	-	-	-
40	134.140	1.00	62.0	149.96	1.00	28.0	155.880	1.00	15.7

* Due to the limitations of commercial software, these conditions are close to ideal, especially on the air side.

**Table 5:** Parameters of the UWCAES system while unloading in selected places for real, unavoidable, and ideal conditions.

State		Real			Unavoidable			Ideal *	
	$\dot{m}$ kg/s	*p*, bar	*T*, °C	$\dot{m}$ kg/s	*p*, bar	*T*, °C	$\dot{m}$ kg/s	*p*, bar	*T*, °C
1–8	-	-	-	-	-	-	-	-	-
9	200.000	14.99	4.0	200.000	14.99	4.0	200.000	14.99	4.0
10	200.000	13.87	148.9	200.000	14.84	114.6	200.000	14.99	100.0
11	200.000	6.16	75.4	200.000	6.13	32.6	200.000	6.09	15.4
12	200.000	5.70	156.1	200.000	6.07	115.2	200.000	6.09	100.0
13	200.000	2.48	75.4	200.000	2.48	32.3	200.000	2.47	15.3
14	200.000	2.29	156.1	200.000	2.45	115.2	200.000	2.47	100.0
15	200.000	1.00	75.7	200.000	1.00	32.4	200.000	1.00	15.5
16–25	-	-	-	-	-	-	-	-	-
26	268.280	1.00	165.0	299.920	1.00	116.9	311.750	1.00	100.0
27–29	-	-	-	-	-	-	-	-	-
30	90.812	1.00	165.0	103.060	1.00	116.9	105.900	1.00	100.0
31	90.812	1.08	165.0	103.060	1.01	116.9	105.900	1.00	100.0
32	90.812	1.00	13.5	103.060	1.00	10.00	105.900	1.00	8.0
33	268.280	1.00	62.0	299.920	1.00	28.0	311.750	1.00	15.7
34	88.735	1.00	165.0	98.430	1.00	116.9	102.930	1.00	100.0
35	88.735	1.08	165.0	98.430	1.01	116.9	102.930	1.00	100.0
36	88.735	1.00	84.9	98.430	1.00	37.1	102.930	1.00	19.4
37	88.735	1.00	165.0	98.430	1.00	116.9	102.930	1.00	100.0
38	88.735	1.08	165.0	98.430	1.01	116.9	102.930	1.00	100.0
39	88.735	1.00	85.3	98.430	1.00	37.2	102.930	1.00	19.7
40	-	-	-	-	-	-	-	-	-

* Due to the limitations of commercial software, these conditions are close to ideal, especially on the air side.

## Data Availability

The data presented in this study are available on request from the corresponding author.
